# Model-Driven Approach for Realization of Data Collection Architectures for Cyber-Physical Systems of Systems to Lower Manual Implementation Efforts

**DOI:** 10.3390/s21030745

**Published:** 2021-01-22

**Authors:** Emanuel Trunzer, Birgit Vogel-Heuser, Jan-Kristof Chen, Moritz Kohnle

**Affiliations:** Institute of Automation and Information Systems, Technical University of Munich, 85748 Garching, Germany; vogel-heuser@tum.de (B.V.-H.); jan.chen@tum.de (J.-K.C.); moritz.kohnle@tum.de (M.K.)

**Keywords:** data collection architecture, data analysis, domain-specific language, IIoT architectures and frameworks, IIoT communication, industrial automation, model-driven development, quantitative evaluation

## Abstract

Data collection from distributed automated production systems is one of the main prerequisites to leverage information gain from data analysis in the context of Industrie 4.0, e.g., for the optimization of product quality. However, the realization of data collection architectures is associated with immense implementation efforts due to the heterogeneity of systems, protocols, and interfaces, as well as the multitude of involved disciplines in such projects. Therefore, this paper contributes with an approach for the model-driven generation of data collection architectures to significantly lower manual implementation efforts. Via model transformations, the corresponding source code is automatically generated from formalized models that can be created using a graphical domain-specific language. The automatically generated architecture features support for various established IIoT protocols. In a lab-scale evaluation and a unique generalized extrapolation study, the significant effort savings compared to manual programming could be quantified. In conclusion, the proposed approach can successfully mitigate the current scientific and industrial challenges to enable wide-scale access to industrial data.

## 1. Integration of Systems and Accessibility of Data as Prerequisites for Industrie 4.0

With the advent of the fourth industrial revolution, called Industrie 4.0 (I4.0), the domain of industrial automation transforms rapidly due to increased digitization of processes and the ever-increasing amount of available data in production. Leveraging this data to adjust machine parameters and production plants is vital for an efficient and flexible production [1,2].

Consequently, a significant prerequisite for the realization of I4.0 is the stronger integration and interconnection of production systems into cyber-physical systems of systems (CPSoS) [3,4,5]. CPSoS are characterized by a large number of distributed systems, distributed control of these systems, partial autonomy, as well as continuous re-configuration and evolution. However, classical automation systems are organized in a hierarchical structure, called the automation pyramid [6], which limits their communication capabilities. The automation pyramid results from divergent boundary conditions on the operational level, with operational technology (OT) to ensure real-time requirements and high reliability, and the superordinate layers that coordinate the production using classical information technology (IT). The long life-cycles of up to 40 years of production plants further complicate this integration as a large number of existing legacy systems need to be supported and their data gathered [7,8,9].

Dotoli et al. [10] conclude that suitable I4.0 technologies for the integration of CPSoS are already available, but significant implementation efforts limit industrial applicability. This can be explained by the significant heterogeneity of existing legacy systems, incompatible communication protocols and interfaces, as well as the large number of different I4.0 technologies currently available [11].

Hence, one of the foremost priorities for adopting I4.0 principles and the increased availability of data are the reduction of the effort to engineer data collection architectures. The term data collection architecture is defined as a system architecture to collect and integrate data from machines in the field as well as from superordinate IT systems for subsequent data analysis [12].

Model-driven development (MDD) can decrease manual implementation efforts significantly [13]. However, as Wortmann et al. [14] point out, there is a lack of adequate metrics and quantitative evaluations in the domain of MDD for I4.0.

Therefore, this paper’s contribution is an approach for the MDD of data collection architectures in the domain of industrial automation. The contribution includes a domain-specific language (DSL) based on the graphical notation by Trunzer et al. [12], as well as a toolchain for the automatic, model-driven generation of the communication architecture of data collection architectures. Furthermore, this paper contributes with a unique extrapolation case study to estimate the generalized efforts savings compared to manual implementation.

The remainder of this paper is structured as follows: in the next section, Section 2, related work and the lack of comparable approaches are discussed. In Section 3, the requirements for an MDD of data collection architectures are discussed (Section 3.1) and the concept for an MDD of data collection architectures is introduced (Section 3.2). The approach is subsequently (Section 4) evaluated in a lab-scale feasibility study and a general extrapolation study, including a mathematical consideration of the effort savings. The paper closes (Section 5) with a conclusion and an outlook on further research in the field.

## 2. Related Work and State-Of-The-Art

The related work is structured into different aspects: after an introduction into Industrial Internet of Things (IIoT) communication protocols (Section 2.1), related approaches for data collection architectures are presented (Section 2.2). Next, an overview of modeling languages and DSLs in general, as well as specific examples, is given (Section 2.3). Finally, the concept of model-driven development is introduced, including relevant state-of-the-art contributions from the field of data collection architectures (Section 2.4). The section closes with a summary of the identified research gap.

### 2.1. IIoT Communication Protocols

Numerous communication protocols and architecture styles are available for IIoT connectivity and system integration [11]. These protocols stem from different domains and have their very own characteristics: while, on the shop floor, OPC UA (OPC Unified Architecture) is propagated as the new de-facto standard for machine connectivity, the Message Queuing Telemetry Transport (MQTT) protocol is predominant in the domain of cheap IIoT sensors. Other protocols encompass the Advanced Message Queuing Protocol (AMQP) protocol with its extensive support of Quality of Service (QoS) features for reliable communication, as well as lightweight and decoupled web technology-based Representational State Transfer (REST) architecture styles. Furthermore, Data Distribution Services (DDS) offer truly decentralized but reliable machine communication and can be found in specific domains, such as robotics or transport systems. An additional alternative is the usage of Apache Kafka and its communication protocol for the high-performance processing of data. An overview and comparison of the different protocols can be found in Trunzer et al. [15].

While some protocols are dominant for specific purposes (e.g., OPC UA on the shop floor or AMQP on higher levels), a full convergence of protocols cannot be foreseen. Therefore, in addition to the heterogeneity of systems and interfaces, the industry is also confronted with heterogeneity in IIoT communication protocols for interoperability and data collection. In addition, the distribution of protocols differs between different domains and applications. Therefore, industry often needs to support more than one communication protocol to be competitive, further increasing the implementation efforts for interoperability and data collection.

### 2.2. Data Collection Architectures

Before being able to analyze and operationalize machine data, it has to be collected from the field. Therefore, special communication structures, so-called data collection architectures, can be used. These are implemented to interface the distributed CPSoS and their superordinate systems to collect all relevant data. This data can include live sensor and actuator signals from the production process and historic measurements, engineering documents, recipes, or orders.

Data-collection architectures must be able to interface the respective systems and access and forward their data to the destination of their processing. Besides syntactic interoperability (structure of data, protocols), semantic interoperability (the meaning of data, information models) has to be ensured to allow all connected systems to process the collected data. Several approaches for data collection architectures and I4.0 system architectures in general that provide data collection functionalities have been published recently.

Gama et al. [16] present a dedicated data collection architecture to collect data from distributed RFID (Radio-frequency Identification) readers. Communication between a central, mediating component and the scanners is established using web service technology. Additionally, the integration of existing legacy systems is not considered.

Peres et al. [17] propose the IDARTS framework, a hybrid multi-agent/Apache Kafka-based architecture for data collection and analysis in industrial automation. While the focus of the architecture is on the field level, superordinate systems could theoretically be interfaced. Nevertheless, the implementation of IDARTS is tailored for the JADE agent framework (Java Agent Development Framework) and Apache Kafka.

In the scope of the COCOP project [18], a plant monitoring architecture is developed. Therefore, data from different hierarchical levels of the automation systems are collected. While different communication technologies (AMQP, OPC UA, and REST) are compared, the implementation is limited to AMQP.

The PERFoRM project [19] proposes an architecture for flexible reconfiguration of CPSoS. PERFoRM foresees the support of various protocols. However, practical implementations are centered around AMQP.

In summary, various data collection architectures can be found in the literature. Still, they are centered around specific protocols and often do not support multiple protocols, limiting their practical applicability.

### 2.3. Modeling Languages and DSLs

Modeling languages allow the abstract description of complex problems. Therefore, model elements are used to describe distinct relevant aspects of systems [20]. Modeling languages consist of an abstract syntax (metamodel), a concrete syntax (notation), and semantics. While the metamodel describes the model elements, their names, and relations, the notation refers to users’ application of the modeling language. The notation can have different representations, for instance, textual or graphical. The semantics relate the model elements to their meaning in the real world.

Their scope of modeling can differentiate modeling languages: while so-called general-purpose modeling languages (GPMLs) can be applied to any domain, DSLs are tailored for specific domains and applications. The loss in universality is compensated by more fine-grained and specific model elements that can allow greater expressiveness of the modeling language.

An example of a relevant modeling language for the description of embedded systems is the UML-profile MARTE (Modeling and Analysis of Real-Time Embedded Systems) [21]. MARTE can be used to model available hardware resources, inputs and outputs (I/Os), and communication interfaces. With this, MARTE is limited to the basic graphical capabilities of UML as it adds no additional visual syntax. In addition, while the internal behavior and dynamics of systems can be modeled, the model elements to capture data flows between complex, interconnected systems are limited. MARTE foresees the specification of properties and requirements related to the systems.

Another example of a modeling language for complex system architectures is the graphical modeling language ArchiMate [22]. While MARTE is focused on a description of low-level hardware interactions, ArchiMate is centered around the description of complex IT architectures with no particular focus on CPSoS. Therefore, model elements for the description of relevant aspects and terms of the industrial automation domain are missing, for instance, master/slave networks or I/O signals.

The group around Vogel-Heuser [12,23,24] presents several variants of a graphical notation for the description of distributed control systems, communication architectures, and data collection architectures. These variants are all based on the same foundation and share the majority of model elements. The graphical notation presented by Trunzer et al. [12] allows the description of systems from the OT up to the IT level, including a separate viewpoint for tracing the flow of data through all systems. However, the basic approach is limited to a graphical notation and does not encompass a metamodel. Therefore, it cannot be considered as a full-fledged DSL. The same group recently published the so-called DSL4hDNCS [25], which features the extension of the graphical notation by a metamodel to allow modeling of heterogeneous distributed, networked control systems (hDNCS) including their time requirements and deployment scenarios.

In conclusion, modeling languages can be used to capture information about complex systems abstractly. While some approaches exist, no graphical DSL for the description of data collection architectures for CPSoS, ranging from OT to IT, can be found in the literature. However, modeling techniques have been identified as key for managing the complexity of CPSoS [4].

### 2.4. State-Of-The-Art in Model-Driven System Architectures

In MDD, models not only serve documentation purposes but are used as essential components during the development of systems. Via so-called model transformations, the modeled information can be leveraged and parts of the development process automated. Model transformations can be differentiated into three kinds: model-to-model (M2M), text-to-model (T2M), and model-to-text (M2T) transformations.

Models can have various shapes, depending on the specific goals and problems that are addressed. For instance, models can be used to formalize or approximate the behavior and characteristics of systems in a mathematical way [26,27], or the structure of systems and software [28].

Some examples of model-driven development of system architectures in general and specifically for data collection can be found in literature. Tekinerdogan et al. [29] developed an approach for the simulation and deployment optimization of DDS systems. Therefore, based on an abstract model of the systems, feasible deployment scenarios are derived automatically and simulated for their feasibility. However, the approach does not contain a graphical modeling language, as well as an automatic set up of the communication infrastructure.

Another relevant approach is the contribution by Benaben et al. [30] related to model-driven engineering of middleware systems. The approach focuses on the domain of enterprise integration between companies. Based on a metamodel, services can be modeled, which are then used to generate web services and configure the middleware components.

Based on the UML MARTE profile, Ebeid et al. [31] introduce a model-driven approach for distributed, embedded systems. The approach captures QoS requirements but is limited to generating runnable configurations for a simulation environment instead of generating code for the embedded systems.

Harrand et al. [32] present the so-called Thing-ML, a textual DSL for embedded IoT devices. The DSL focuses on the description of embedded devices and low-level interactions between them. Based on the models, basic communication interfaces can automatically be generated.

Thramboulidis and Christoulakis [33] propose a model-driven development of microservice architectures for CPSoS based on a metamodel. The approach aims to replace existing infrastructures and does not take legacy systems into account. In addition, the modeled level of detail concerning the hardware, software, and networks is relatively low. The approach does not include the means to model data flow between systems. It is limited to the non-graphical presentation using the metamodel and a single, propriety protocol.

Another approach is presented by Mazak et al. [34] based on an extended version of the generic, non-graphical exchange format AutomationML (AML). The authors extend AML with a description of data dependencies between systems. Based on the modeled information, OPC UA servers act as data providers and a data collection architecture, including a central data repository, are automatically set up. The approach is tailored for greenfield environments without special consideration of legacy systems. The flow of data are implicitly modeled over the introduced dependencies, but it cannot be followed through the system or over a multi-stage process.

While MDD is an accepted paradigm for software development, only a limited number of approaches can be found for MDD of data collection architectures. None of the presented approaches includes a graphical DSL. Furthermore, none of the approaches provides support for multiple communication protocols and includes considerations related to legacy systems.

### 2.5. Identified Research Gap

While several system architectures for data collection and related modeling approaches exist, no comprehensive graphical DSL for the description of data collection architectures, nor an integrated, model-driven approach for automatic generation and setup of the communication infrastructure can be found literature. In addition, as Wortmann et al. [14] pointed out, there is a significant lack of quantitative evaluations and proofs for implied efforts savings when using MDD in the domain of I4.0.

## 3. Requirements and Concept for a Model-Driven Data Collection Architectures

In the following section, the requirements for a model-driven data collection architectures are presented. Afterwards, the derived concept for and integrated, model-driven development of data collection architectures is given.

### 3.1. Requirements for a Model-Driven Development of Data Collection Architectures

To be practically applicable, an approach for MDD of data collection architectures has to fulfill several requirements (indicated by *R*), which are derived below:

*R_DSL_*  During data collection and analysis projects in the domain of industrial automation, a multitude of disciplines is involved, ranging from data analysts, over IT architectures, to automation engineers and process experts. All of these experts have different backgrounds and use different terminology. Therefore, the approach should be based on a graphical DSL that allows for sharing modeled information intuitively and understandably. Moreover, the graphical notation and the underlying metamodel have to provide the means to capture all relevant aspects needed for the design of data collection architectures in the domain of industrial automation.
*R_Com_*  The realization of data collection architectures requires the repetitive implementation of numerous communication channels that form the communication part of the architecture, and specific glue code that transforms, analyzes, or stores the collected data. The approach should be capable of automatically generating code for the communication part to reduce manual implementation efforts efficiently.
*R_Prot_*  The significant heterogeneity of IIoT protocols and the uncertainty, whose protocol will be predominant in the future, hinders and slows down industrial adoption of I4.0 principles. In addition, if a product has to be offered for different markets or domains, manufacturers may have to support multiple protocols for the same machine. This further increases the implementation efforts for data collection architectures. Therefore, the approach should feature modular support for relevant IIoT protocols to increase technology adoption.
*R_Init_*  Initial deployments (development from scratch) of data collection architectures are associated with substantial implementation efforts due to heterogeneity and complexity of CPSoS. MDD has the potential to significantly lower these efforts in comparison to manual programming. However, these efforts’ savings have to be quantifiable and of general validity and not just of qualitative nature, as found in the literature. The quantification has to take the effort to create the MDD toolchain into account to estimate the break-even between MDD and classical manual programming of data collection architectures.
*R_Mig_*  Besides initial deployment, re-deployments, also called migrations, are of significant interest for industrial applicability [10]. Despite the availability of new solutions, which are better suited for the needs of automated production, enterprises hesitate to apply them. This is due to the excessive cost associated with re-implementing all connected systems’ communication interfaces, consequently causing vendor lock-in. In addition, the need to support more than one communication protocol for data collection inside CPSoS can be seen as a migration scenario. This leads to the requirement that the MDD approach has to support migration scenarios of data collection architectures. In addition, effort savings also have to be proofed quantitatively for such a scenario compared to manual programming.

### 3.2. Concept for Model-Driven Data Collection Architectures

**Figure 1:**
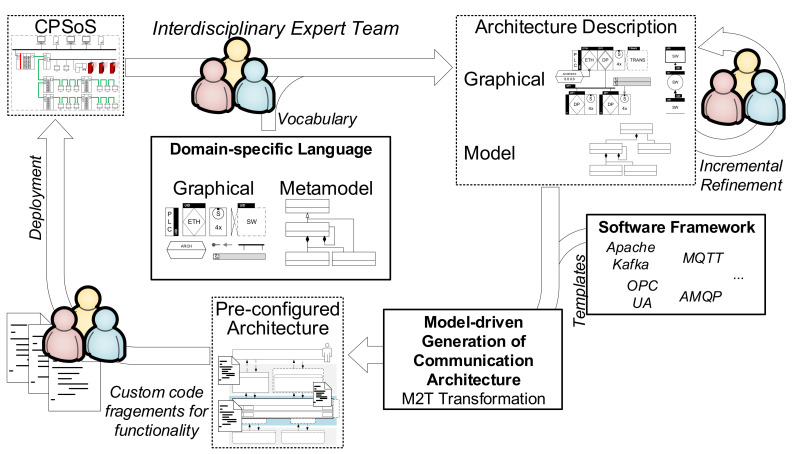
Workflow for the model-driven development of data collection architectures.

In the following section, the proposed concept for an MDD of data collection architectures is presented. Figure 1 summarizes the proposed workflow. Based on an existing or intended CPSoS, an interdisciplinary expert team designs and models the CPSoS and the associated data collection architecture using a graphical DSL (Section 3.2.1). The resulting architecture description is made up of a human-readable graphical representation and a computer-readable model containing modeled information. After incremental refinement of the architecture description by the involved disciplines, a model-driven generation of the communication part of the data collection architecture is carried out (pre-configured architecture, Section 3.2.3). Therefore, an M2T transformation transforms the architecture model into code using templates from a library (Section 3.2.2). The pre-configured architecture is then extended by the expert team with custom glue code that transforms, manipulates, or stores data. After this step, the data collection architecture can be deployed to the field.

#### 3.2.1. Graphical Notation and Metamodel of the DSL

**Figure 2:**
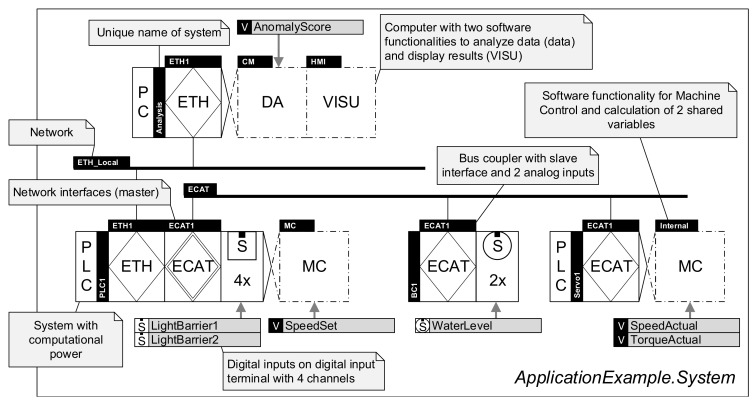
Application example of the visual notation of the DSL in the system viewpoint.

**Figure 3:**
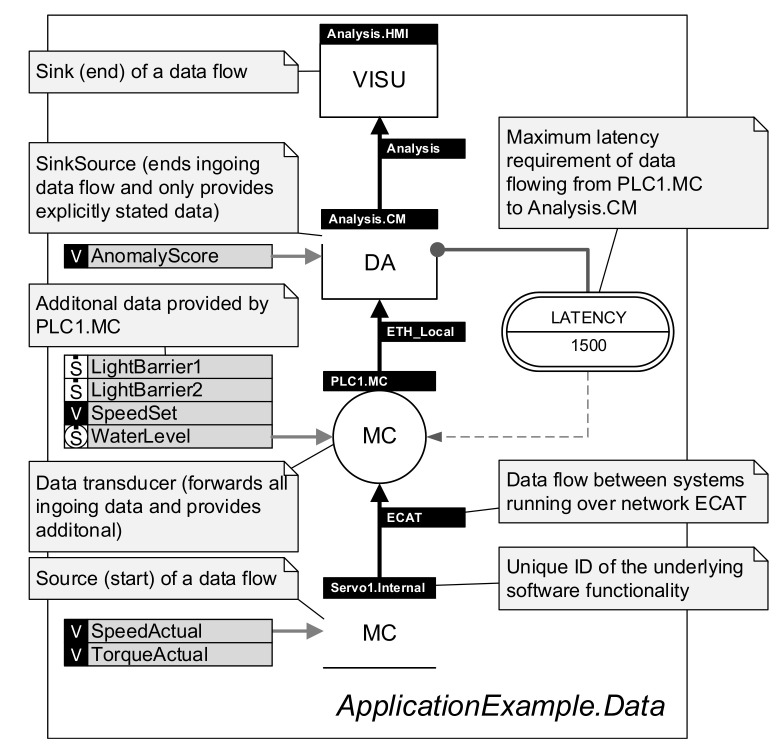
Application example of the visual notation of the DSL in the data flow viewpoint.

The graphical notation presented by Trunzer et al. [12] serves as a basis for the proposed DSL ($R_{DSL}$). It contains all relevant model elements to describe data collection architectures for CPSoS. In Figure 2 and Figure 3, a short, annotated application example demonstrating the modeling capabilities of the notation is given. For a full introduction to the model elements of the graphical notation, the reader is referred to the original source.

The notation includes two distinct viewpoints to model data collection architectures: first, a system viewpoint (cf. Figure 2) to characterize the hardware of CPSoS, software executed as part of it, and the locations where data are generated. Second, a data flow viewpoint (cf. Figure 3) that reflects the flow of data through the systems and the role of each subsystem. Both viewpoints are linked using a unique labeling system to identify all related systems. Furthermore, the notation allows specifications of several properties and requirements, including time characteristics, data properties, and architectural aspects.

**Figure 4:**
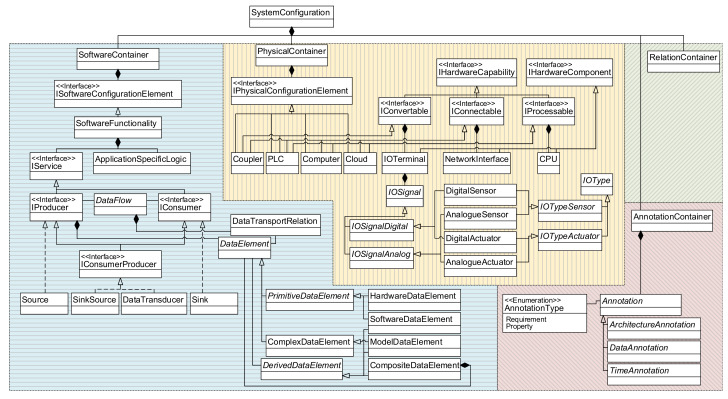
Overview of the metamodel (blue: *SoftwareContainer*, yellow: *PhysicalContainer*, green: *RelationContainer*, and red: *AnnotationContainer*.

As the graphical notation only describes the DSL’s concrete syntax, it is supplemented by a metamodel (abstract syntax). Modeled information is stored as an instance of the metamodel (a model), with the metamodel describing the rules of how model elements can be combined and linked. An overview of the metamodel is given in Figure 4 as an excerpt of the whole metamodel. It is divided into four main *Containers* that are aggregated to describe a *SystemConfiguration*:

- a *SoftwareContainer* for the description of data flows and software functions,
- a *PhysicalContainer* that reflects the hardware systems and components of the architecture,
- an *AnnotationContainer* that can carry additional information on properties and requirements, as well as
- a *RelationContainer* to describe logical links.

Inside the *SoftwareContainer*, the actions carried out by software systems are differentiated in communication functionalities (derived from *IService)* and the logic that works with the data (glue code, e.g., data manipulation) as *ApplicationSpecificLogic*. IServices connect various *SoftwareFunctionalities* over so-called *DataFlows*. Here, every *DataFlow* can transport *DataElements* as a payload (see *DataTransportRelation*).

The *PhysicalContainer* contains a description of the hardware systems and components that constitute the overall system. Complex systems, e.g., programmable logic controllers (*PLCs*) can be composed of several hardware components, for instance *IOTerminals* or *NetworkInterfaces*. To decouple the composition logic from the actual hardware components, capabilities are introduced over the interface *IHardwareCapability*. This allows an easy extension of the metamodel if new hardware components are needed and simplifies the system description. Actual hardware signals (*IOSignals*) are aggregated by *IOTerminals*. Following the graphical notation, these signals are differentiated by their type of signal (digital/analog) and the component (sensor/actuator).

**Figure 5:**
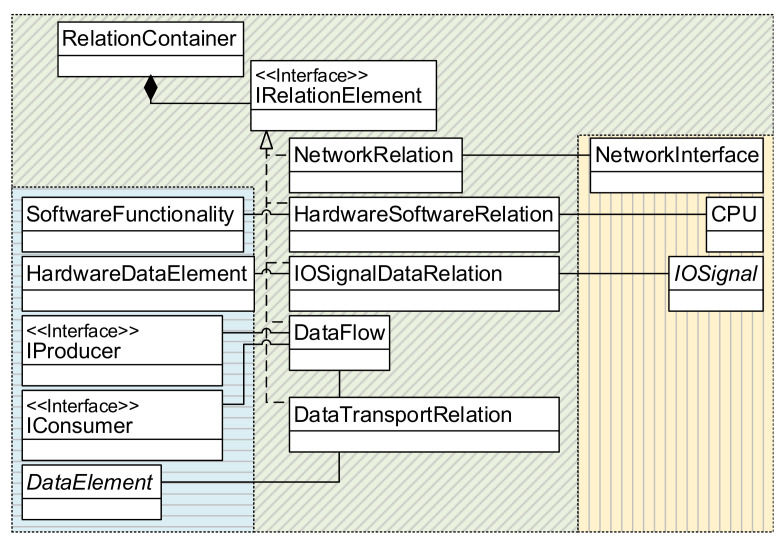
Mapping of software and hardware elements using the RelationContainer.

While *Annotations* from the *AnnotationContainer* can carry additional information as seen in the application example, the *RelationContainer* (see a detailed view in Figure 5) mainly links elements from the physical and the software containers. It is used to associate hardware signals with the related variables in software or describe which software runs on which hardware system.

While the metamodel of the DSL is realized in the Eclipse Modeling Framework (EMF) (https://www.eclipse.org/modeling/emf/, accessed 16 December 2020). As the de-facto standard for MDD, the graphical notation is provided as stencils for Microsoft Visio (https://www.microsoft.com/de-de/microsoft-365/visio/flowchart-software, accessed 16 December 2020). An automatic link and synchronization between the graphical representation and the model instance are currently not part of the implementation, but can be realized inside the Eclipse ecosystem relatively easy with Graphiti (https://www.eclipse.org/graphiti/, accessed 16 December 2020) or Sirius (https://www.eclipse.org/sirius/overview.html, accessed 16 December 2020).

#### 3.2.2. Software Framework for IIoT-Protocol Support

A modular software framework with support for various IIoT communication protocols complements the approach. While the framework can be used independently of the proposed MDD approach, it provides the necessary code templates for the model transformation step.

The heart of the framework is the definition of standardized interfaces that abstract the basic functionalities of the communication protocols, such as publishing data or subscription to updates. On the application side, programmers use these generic functionalities for sending and receiving data without the need to know the backing communication technology. The related technology-specific functionality is then implemented as part of the software framework and hidden behind the standard interfaces. In addition, due to the modular design of the framework, additional communication protocols can be supported via the interface’s implementation.

Legacy systems that do not support the software framework or the communication protocols can be interfaced using so-called data adapters. These adapters are placed between the legacy system and the data collection architecture and translate syntax and semantics of the in- and outgoing data to ensure interoperability. While the communication code on the data collection side can be generated automatically, not only the glue code that translates the data but also the communication code for interaction with the legacy device has to be coded manually. However, this offers the possibility to also interface exotic or unsupported systems.

The software framework is realized in C# and .NET Core 3.1 (https://github.com/dotnet/core, accessed 16 December 2020)—for cross-platform support, including Windows, macOS, and Linux. As part of the software framework, several technology-specific implementations are currently provided with support for the established IIoT protocols AMQP, Apache Kafka, MQTT, and OPC UA ($R_{Prot}$). Furthermore, interoperability with other programming languages and frameworks is established by providing a defined gRPC (https://grpc.io/, accessed 16 December 2020) interface. gRPC is a high-performance remote procedure call (RPC) framework from Google that is available for a multitude of programming languages. For instance, the software framework features additional support for the Beckhoff (Automation Device Specification) protocol using the gRPC interface, as the necessary ADS libraries are not available for the .NET Core framework, but instead only for the .NET Framework 4.0.

#### 3.2.3. Model Transformation to Deployable Communication Code

In the last step, the instances of the DSL’s metamodel serve as a basis for the automatic generation of the communication part of the data collection architecture via an M2T transformation. Therefore, code templates with placeholders containing system-specific information (e.g., communication protocol, IP addresses, ports, variables to be collected) from the software framework are combined and filled based on the modeled information ($R_{Com}$). The glue code that handles the data are not part of the automatic generation. Still, protected placeholder sections are generated, where programmers may add functionality to the architecture. These custom code fragments are preserved in case of a regeneration of the communication architecture. As the generated code is based on the software framework with its standard interfaces, programmers do not need to worry about communication technologies and can rely on the standard interface, therefore ensuring interoperability and compatibility with any implemented communication technology of the software framework. In addition, the transformation engine has a minimum set of rules to check the consistency of the modeled information.

While the presented transformation relies on the software framework and C#, a model based on the proposed DSL can theoretically be transformed to any other programming language, leading to an independence of model and specific implementation.

Acceleo (https://projects.eclipse.org/projects/modeling.m2t.acceleo, accessed 16 December 2020), as an implementation of the OMG’s MOF model to text transformation language [35], is used for the model transformation step.

## 4. Evaluation of the Model-Driven Approach for Data Collection Architectures

The evaluation of the proposed approach for MDD of data collection architectures is carried out in two case-studies: at first, a sufficiently complex lab-scale scenario is used to compare implementation efforts for the implementation of data collection architectures for CPSoS. Afterward, an extrapolation case study tries to generalize the assumed effort savings as a function of the relevant boundary conditions and is complemented by a border case analysis. Both case-studies try to assess the effort savings using the proposed approach based on a worst-case assessment.

### 4.1. Lab-Scale Case-Study to Investigate Implementation Effort Savings

In the lab-scale case-study, a complex CPSoS with different hardware components and support of communication protocols that is connected with superordinate IT systems for data analysis, monitoring, and visualization is considered. Therefore, a data collection architecture is needed to collect and forward the relevant information from the CPSoS to the other systems. For the case-study, the AMQP protocol was chosen for the initial realization of the data collection architecture. To compare the efforts of manual implementation and the proposed MDD, the same architecture will be implemented twice: on the one hand, the architecture is modeled using the proposed DSL and then transformed into code. On the other hand, a minimal data collection architecture with the same base functionality is implemented by hand. This evaluation will focus on the communication part of the data collection architecture. It is assumed that the glue code for both cases is comparable and, hence, of the same complexity.

#### 4.1.1. Description of Use-Case Featuring a Heterogeneous CPSoS with Superordinate Systems

**Figure 6:**
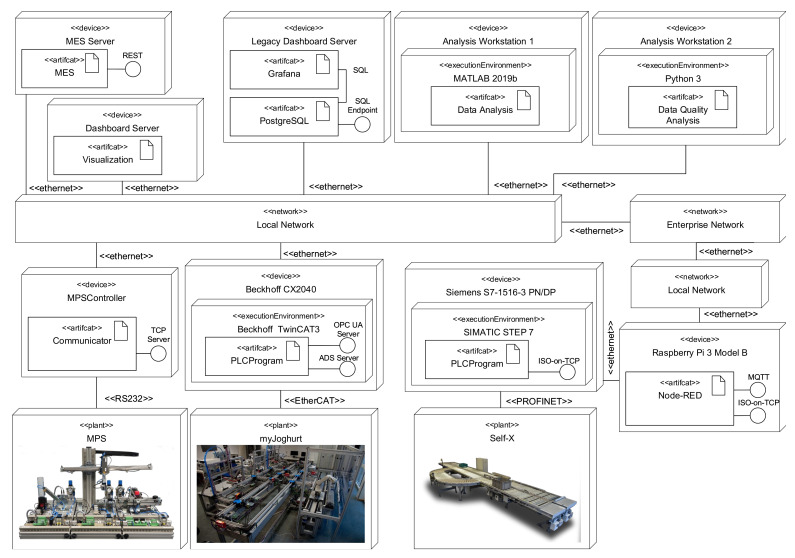
Connected systems of the lab-scale case-study without gateways and infrastructure components as a UML deployment diagram.

An overview of the considered systems is given in Figure 6 as a UML deployment diagram. The case-study encompasses three distinct and representative Cyber-physical Production Systems (CPPS):

1. the legacy CPPS Festo Modular Production System (MPS) that is interfaced using custom software that provides connectivity over a proprietary TCP protocol and communicates with the plant over a serial RS232 connection;
2. the constantly evolving myJoghurt Industrie 4.0 demonstrator, with a state-of-the-art Beckhoff PLC, connectivity over the proprietary Beckhoff ADS protocol as well as standard OPC UA, and around a total of 500 variables (I/Os and internal variables); and
3. the Self-X material flow demonstrator [36] equipped with a Siemens S7-1500 PLC, Munich, Germany, around 170 I/Os, and the support for the Siemens ISO-on-TCP protocol [37].

While the MPS and the myJoghurt plant are directly connected to an internal Ethernet network, the Self-X demonstrator is located in another Ethernet network with a Node-RED-based (https://github.com/node-red/node-red, accessed 16 December 2020). gateway that translates between the ISO-on-TCP protocol and standard MQTT.

In addition, several superordinate systems are part of the case-study, including data analyzers, dashboards for visualization, a mocked-up Manufacturing Execution System (MES), and data storage systems. The actual realization of the data collection architecture will encompass additional systems that act as infrastructure components for data transportation or transformation.

#### 4.1.2. Model-Driven Generation of Communication Architecture

Using the graphical DSL, the data collection architecture was modeled. Additional infrastructure components, e.g., data adapters and the AMQP message broker, were conceptualized and modeled to yield a complete model of the architecture. The case-study foresees several interwoven data flows between systems to reflect complex scenarios. For instance, data from the myJoghurt plant are analyzed together with order data to calculate performance key performance indicators (KPIs). These are then forwarded together with the raw data to the monitoring dashboard. The full graphical models of the case-study, including a full description of all connected and infrastructure components, as well as all data flows, and additional annotations can be found in the supplementary material.

For better comparability, the conceptualized architecture was modeled by three persons after a short introduction to the DSL. Table 1 summarizes the time efforts for modeling $E_{MDD,Init}$. The measured values include the time for layout adjustments and consistency checks. For the case-study, the worst modeling time of person 3 was chosen as a baseline to assess the approach in a worst-case scenario (high modeling effort).

After the modeling step, the model-transformation was executed, generating an AMQP-based data collection architecture. The generation includes the C# code with placeholders for custom code, the project files for Visual Studio that include references to required libraries, as well as configuration files for Docker and the RabbitMQ broker (https://www.rabbitmq.com/, accessed 16 December 2020). In total, 4284 lines of C# code were generated, as well as 616 lines of configuration and project files. In the next step, the glue code was implemented manually, and the code was compiled and deployed to the respective systems. The deployed data collection architecture was fully functional, and all modeled data flows were working and transporting data correctly.

#### 4.1.3. Effort Metrics for Initial Deployment

In parallel, the data collection architecture was entirely implemented by hand using C# and the same libraries to replicate the exact modeled functionality of data collection. As the manually programmed code can focus on a minimal approach with lower modularity and, therefore, code overhead, only 989 lines of code ($LoC_{Man}$) were needed to realize the data collection functionality. Afterward, glue code was implemented, and the architecture was compiled and deployed, once again resulting in a working data collection architecture.

To compare the implementation efforts for the communication part of the data collection architecture between the proposed model-driven and the manual programming approaches, the programming effort needs to be quantified. Therefore, productivity metrics for experienced programmers were collected from the literature. While Cusumano and Kemerer [38] report an average productivity $p_{LoC}$ of 436 lines of code (LoC) per programmer and month in Europe, Prechelt [39] reports a productivity of 36 LoC/h (approx. 6200 LoC/month) for the upper quartile using Java as programming language (very similar to C#). The studies’ different scopes can explain the variations: While Cusumano and Kemerer investigate 104 large-scale projects written in different programming languages and including project-related tasks (e.g., testing, documentation), Prechelt focuses on smaller projects executed by a single programmer. It can be concluded that LoC metrics for programmer productivity deviate significantly and have to be treated with caution.

However, for a worst-case assessment of the proposed approach, in the following, the exceptionally high productivity reported by Prechelt will be used to assess the worst-case performance of the approach. Nonetheless, in future studies, more sophisticated metrics, such as COCOMO II [40], should be employed to increase the accuracy of the effort comparison.

With $p_{LoC}=36\;\mathrm{LoC}/h$, the programming effort for a manual, initial implementation $E_{Man}$ equals

(1) $$\begin{array}{c} E_{Man}=\frac{LoC_{Man}}{p_{LoC}}=27.47\;h. \end{array}$$

Therefore, the relative effort $r_{E}$ of the proposed model-driven approach compared to manual software programming is

(2) $$\begin{array}{c} r_{E}=\frac{E_{MDD}}{E_{Man}}=16.96\%. \end{array}$$

It can be concluded that, even under a worst-case scenario, the effort savings for an initial deployment of the data collection are significant when using the proposed approach ($R_{Init}$). When assuming an average hourly cost of 50 US$ for a programmer, a total of about 1140 US$ could be saved even saved for the relatively small lab-scale case-study. In case of a migration, major parts of the manually programmed communication code would require significant refactoring. At the same time, changes for the model-driven approach are limited to an update of the protocol annotations and subsequent repetition of the model transformation step. In the following section, a generalized extrapolation study is carried out with a special consideration and quantification of effort savings on dependence of migrations.

### 4.2. Extrapolation Case-Study for a Generalization of Results

The extrapolation case-study aims to generalize the previous findings and also, in contrast to the lab-scale case-study, take the effort for the development of the DSL and the model-driven approach into account. Hence, it tries to answer the questions, how large a data collection architecture (or several realizations using the same toolchain in sum) would have to be in order to justify the development of the model-driven approach. Therefore, the study considers the total effort as a function of the number of publisher/subscriber pairs $N_{Pair}$ and the average number of variables exchanged per pair ${\bar{N}}_{Var}$. In the following, the relations between $N_{Pair}$, ${\bar{N}}_{Var}$, and the efforts $E_{Man}$ and $E_{MDD}$, as well as the effort ratio $r_{E}$, are given, including a mathematical analysis. Afterwards, the break-even between both approaches is investigated based on different scenarios.

#### 4.2.1. General Analysis of the Relations

In general, both efforts $E_{i}$ are functions of $N_{Pair}$ and ${\bar{N}}_{Var}$. For the manual implementation, the implementation effort $E_{Man}$ depends on the total amount of code and the programmer’s productivity if the efforts can be assumed as constant for each pair and each variable

(3) $$\begin{array}{c} E_{Man}=\frac{LoC_{Man}}{p_{LoC}}. \end{array}$$

The lines of code for a manual implementation can be calculated as

(4) $$\begin{array}{cc} LoC_{Man} & =N_{Pair}\;\cdot \;LoC_{Pair}+N_{Pair}\;\cdot \;{\bar{N}}_{Var}\;\cdot \;LoC_{Var}, \end{array}$$

with $LoC_{Pair}$ the lines of code for implementing of a publisher/subscriber pair and $LoC_{Var}$ the additional lines of code for every exchanged variable in every pair.

In contrast, the total effort for the model-driven realization of a data collection architecture $E_{MDD}$ corresponds to

(5) $$\begin{array}{c} E_{MDD}=E_{Model}+E_{MDD,Init}=E_{Model}+\left(E_{Framework}+E_{M2T}+E_{DSL}\right), \end{array}$$

with $E_{Model}$ is the effort to model the systems and $E_{MDD,Init}$ the initial effort for the creation of the model-driven toolchain. $E_{MDD,Init}$ can be decomposed into the programming effort for the creation of the software framework $E_{Framework}$ that provides the template for the M2T step, the programming effort for the M2T transformation $E_{M2T}$, as well as the development effort for the DSL including full tool support $E_{DSL}$.

Furthermore, the modeling effort can be expressed as

(6) $$\begin{array}{c} \begin{array}{cc} E_{Model} & =N_{Pair}\;\cdot \;e_{Pair}+N_{Pair}\;\cdot \;{\bar{N}}_{Var}\;\cdot \;e_{Var}, \end{array} \end{array}$$

with $e_{Pair}$ the time needed for the modeling of a publisher/subscriber pair and $e_{Var}$ the time effort for modeling of a transported variable.

Therefore, the general effort ratio $r_{E}$ can be expressed as

(7) $$\begin{array}{c} \begin{array}{cc} r_{E} & =\frac{E_{MDD}}{E_{Man}}\\ \; & =\frac{N_{Pair}\left(e_{Pair}+{\bar{N}}_{Var}\;\cdot \;e_{Var}\right)+E_{MDD,Init}}{\frac{1}{p_{LoC}}\;\cdot \;N_{Pair}\left(LoC_{Pair}+{\bar{N}}_{Var}\;\cdot \;LoC_{Var}\right)}. \end{array} \end{array}$$

In general, it can be stated that the set {r|r<1} is a subset of the image of $r_{E}$. This allows for deducing the theoretical existence of a more efficient realization by the model-driven approach. The effect of the system size on $r_{E}$ is further investigated to provide generally valid statements on the effectiveness of the model-driven approach. For this, the gradient of $r_{E}$

(8) $$\begin{array}{c} \nabla r_{E}\left(N_{Pair},{\bar{N}}_{Var}\right)=\left(\begin{array}{c} \frac{\partial r_{E}}{\partial N_{Pair}}\\ \frac{\partial r_{E}}{\partial {\bar{N}}_{Var}} \end{array}\right)=\left(\begin{array}{c} -\frac{E_{MDD,Init}}{N_{Pair}^{2}\;\cdot \;\frac{1}{p_{LoC}}\;\cdot \;\left(LoC_{Pair}+{\bar{N}}_{Var}\;\cdot \;LoC_{Var}\right)}\\ p_{LoC}\;\cdot \;\frac{e_{Var}\;\cdot \;LoC_{Pair}-LoC_{Var}\;\cdot \;\left(e_{Pair}+\frac{E_{MDD,Init}}{N_{Pair}}\right)}{{\left(LoC_{Pair}+{\bar{N}}_{Var}\;\cdot \;LoC_{Var}\right)}^{2}} \end{array}\right) \end{array}$$

is used. Moreover, it is assumed that the systems contain elements ($N_{Pair}$ and ${\bar{N}}_{Var}>0$) and these elements require effort. Accordingly,

(9) $$\begin{array}{c} \frac{\partial r_{E}}{\partial N_{Pair}}<0 \end{array}$$

is valid if $E_{MDD,Init}$ is considered. With these assumptions, the ratio function falls strictly monotonously in the dimension $N_{Pair}$. By also considering the limit value of $r_{E}$ for increasing $N_{Pair}$

(10) $$\begin{array}{c} \lim_{N_{Pair}\to \infty }r_{E}=\frac{e_{Pair}+{\bar{N}}_{Var}\;\cdot \;e_{Var}}{\frac{1}{p_{LoC}}\left(LoC_{Pair}+{\bar{N}}_{Var}\;\cdot \;LoC_{Var}\right)} \end{array}$$

and the strict monotony mentioned before, it can be concluded that a model-driven approach leads to savings in effort when increasing $N_{Pair}$ if

(11) $$\begin{array}{c} \left(e_{Pair}+{\bar{N}}_{Var}\;\cdot \;e_{Var}\right)<\frac{1}{p_{LoC}}\left(LoC_{Pair}+{\bar{N}}_{Var}\;\cdot \;LoC_{Var}\right). \end{array}$$

#### 4.2.2. Break-Even Analysis between Manual Implementation and the Model-Driven Approach

After the derivation of the correlations in the previous section, a break-even analysis is carried out. Therefore, in the first step, representative LoC metrics for a publisher/subscriber pair as well as for a transported variable have to be determined. All protocols supported by the software framework are analyzed and minimal, but modular functional code samples are programmed. The respective LoC per protocol is determined and decomposed in LoC per pair and variables. Afterward, the mean number of LoC across all supported protocols is chosen as a representative metric. The minimal code samples try to define reusable methods to decrease the number of LoC that need rework during migrations from one protocol to another.

Table 2 summarizes the findings. Lines that include authentication details are not counted as these also will have to be adjusted for the automatically generated code from the MDD toolchain. Furthermore, for the calculation of the effort $E_{Man}$, the productivity $p_{LoC}$ of 36 LoC/h is assumed here as well. All code samples can be found in the supplementary material, as well as in a public git repository (https://gitlab.lrz.de/TUMWAIS/public/datacollectionarchitecture.modeldriven).

In the next step, the modeling effort and the effort to create the toolchain have to be determined. Therefore, the minimal model that corresponds to the code samples is modeled by hand. The specific efforts $e_{i}$ measured for an experienced engineer can be found in Table 3.

For the estimation of effort for the toolchain creation, the following figures have been determined: the software framework encompasses a total of approx. 4000 LoC, which yields with $p_{LoC}$ an effort $E_{Framework}$ of 111 h. The M2T transformation implemented in Acceleo has a total of 1350 LoC. Under the assumption that $p_{LoC}$ is also valid for Acceleo code, this corresponds to $E_{M2T}=38\;h$. Finally, the effort for the development and tool development of the DSL is estimated to a full person-year ($E_{DSL}=2078\;h$).

**Figure 7:**
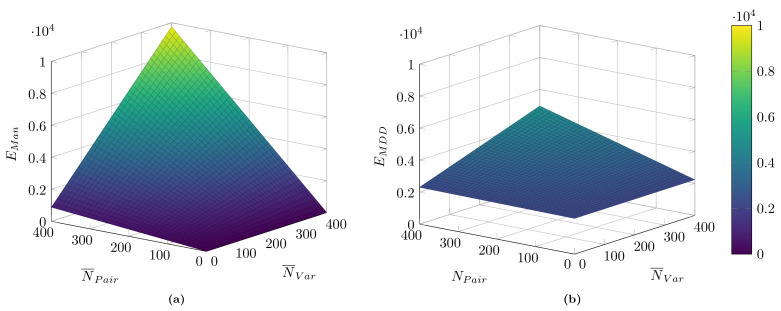
Comparison of implementation efforts for an initial deployment of a data collection architecture as a function of the number of publisher/subscriber pairs and the average number of variables per pair. Scenarios encompass an initial deployment and without a subsequent migration. (**a**) manual implementation; (**b**) MDD approach.

With these parameters, the efforts $E_{i}$ for both development approaches of data collection architectures can be calculated as functions of $N_{Pair}$ and ${\bar{N}}_{Var}$. An example is depicted in Figure 7 for the case of an initial deployment without any additional migration. As can be seen, especially for small systems, the manual approach outperforms the model-driven approach due to the additional effort for the toolchain creation. Nevertheless, the model-driven effort increases significantly slower for both increasing $N_{Pair}$ as well as ${\bar{N}}_{Var}$ in comparison with the manual approach. Therefore, for large systems or the realization of several independent data collection architectures based on the same toolchain, a break-even can be expected that justifies the development of the toolchain.

**Figure 8:**
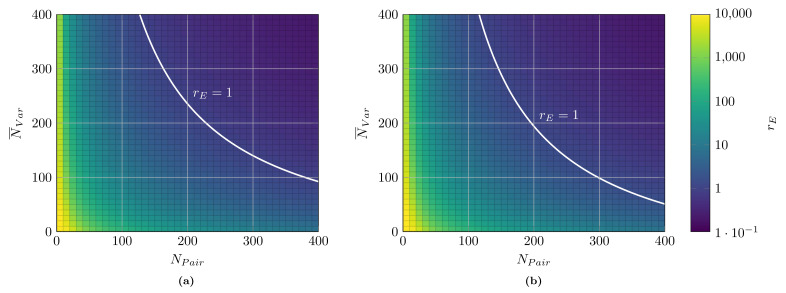
Relative effort $r_{E}$ between MDD of data collection architectures and classical, manual programming with break-even ($r_{E}=1$) marked. Log-scale of colormap. (**a**) only initial; (**b**) initial + one migration

Figure 8 represents the relative efforts $r_{E}$ for two distinct scenarios: firstly, only an initial deployment (cp. Figure 7); and, secondly, initial deployment and one subsequent migration of the data collection architecture to another protocol. As expected, the architecture’s manual programming is strongly to prefer compared to an MDD approach for small systems. Here, the effort for the toolchain creation does not pay off. However, with an increasing number of systems (pairs) and transported information (variables), $r_{E}$ decreases strongly. For the case of the initial deployment (cp. Figure 8a), a break-even ($r_{E}=1$) can be observed. For instance, for systems with 300 pairs and 140 variables each (on average), the implementation efforts are the same (here, assuming the average hourly cost of a programmer of 50 US$ relating to a total of approx. 165,000 US$ implementation costs). After this, for even larger systems, the proposed approach strongly outperforms manual programming ($R_{Init}$).

If migrations are considered (cp. Figure 8b), the relative efforts decrease, and MDD is viable for even smaller systems. For instance, for a system with 300 pairs and 99 variables per pair, $r_{E}$ of 1 is reached when an additional migration is included (relating to a total implementation cost of 139,000 US$). The more migrations are planned (or, the more different variants with support for different protocols have to be supported in parallel), the better the proposed approach’s performance is in comparison to classical software programming ($R_{Mig}$).

In summary, the conservative extrapolation case-study demonstrated the performance of the proposed approach for the MDD of data collection architectures in general. While for small systems, a classical software development approach is strongly preferred, for larger projects, the proposed approach strongly outperforms classical programming. Since the developed toolchain can be reused for several, independent realizations and the determined numbers are realistic regarding the sizes of the systems required for a break-even for industrial scenarios, the proposed approach can represent a feasible alternative for practical realizations.

## 5. Conclusions and Outlook

Data collection from heterogeneous CPSoS is one of the main prerequisites for the practical realization of I4.0 concepts. Only when data are widely available can they be used to provide additional insights. However, the manual development of data collection architectures for CPSoS is time-consuming and expensive due to the wide variety of available protocols and legacy systems.

Therefore, this work contributed with an innovative and integrated, model-driven approach for the automatic generation of the communication part of data collection architectures. Based on a graphical DSL, complex data collection scenarios in CPSoS can be described in collaboration with several involved disciplines. The models then serve as the basis for the model transformation to yield a functional data collection architecture. The proposed approach currently supports a multitude of established IIoT protocols. In a lab-scale case-study, the approach proved its feasibility and demonstrated significant effort savings compared to classical software programming. In a unique extrapolation study, this contribution tries to address the research gap identified by Wortmann et al. [14] and provides a general, quantitative evaluation of the approach. A significant saving of efforts could also be proved for generalized use-cases. Furthermore, it could be demonstrated that, for larger systems in the size of industrial systems, the development of the underlying toolchain for the MDD of data collection architectures can be justified by the effort savings.

Future work is dedicated to several aspects: on the one hand, an extended extrapolation case-study using a sophisticated effort estimation model such as COCOMO II instead of LoC metrics could increase the validity of the results. On the other hand, the approach would benefit from better integration into the engineering process. As several artifacts describe, for instance, bus configurations and signals are available in the respective engineering tools, e.g., Siemens TIA, reusing this information would relieve the experts from remodeling this information, remove redundancies, and further increase the expected efforts savings compared to manual programming. A fully-integrated modeling environment with bi-directional synchronization between graphical representation and the model instance is vital for industrial applications from a practical point of view. Nevertheless, from a scientific point of view, this will not alter the achieved results. As always with systems composed of various, heterogeneous systems, the trust between all participating systems is a significant concern [41]. Malicious systems could, for instance, purposely send incorrect data or try to disturb data transfer inside the network (e.g., (Distributed) Denial of Service (DDoS) attacks). Including these aspects into the DSL and provide means to monitor the correct operation of the CPSoS could be a valuable extension of the approach.

Besides trust, the extension of the approach for capturing the field level in more detail would be very relevant. For instance, the metamodel of the DSL4hDNCS by Vogel-Heuser et al. [25] has the same basis as the one contributed in this work. In addition, the graphical notations are already fully aligned. By consolidating the modeling approaches and their metamodels, a wide range of OT/IT-systems could be modeled, including control aspects, data generation, and data collection. This could also provide valuable insights into factors such as timing behavior of systems, the influence of different fieldbus or network configurations (e.g., local area networks vs. remote computing over wide area networks), and the deployment of software for data collection onto resource-constrained control devices. The models could also be used to find feasible deployment alternatives and optimize the overall configuration of the CPSoS. Furthermore, extending the approach to also capture not only the dynamics of systems, but of data and its characteristics could close the gap between collection and processing of data.

An additional interesting aspect is the transfer and application of the proposed concepts for IoT environments. Here, distinct requirements, such as limited computational power and communication links, as well as the interaction with low-level electronics will require modifications and extensions to the metamodel, as well as the model-transformation step. Moreover, the provided code temples have to be replaced to support established toolchains for embedded devices (e.g., provided as C-Code). Here, the heterogeneity in available controllers, their programming interfaces and interaction with registers, as well the substantial limitations put on the communication libraries concerning available memory, need special considerations. However, the proposed approach can also enable significant effort savings in the realization of IoT scenarios and possibly easy integration into IIoT environments.

## Figures and Tables

**Table 1:** Modeling efforts $E_{MDD,Init}$ for modeling the lab-scale case-study of three persons and their experience level with the notation and background in industrial automation.

Person	Experience Level	Total Effort for Modeling of the Lab-Scale Setup
1	Well-experienced user, strong industrial automation background applied the graphical notation several times.	2 h 30 min
2	Semi-experienced user, medium industrial automation background, applied the notation occasionally.	4 h 20 min
2	Inexperienced user, strong industrial automation background, recently introduced to the notation.	4 h 40 min

**Table 2:** Manually programmed lines of code for a minimal producer and subscriber pair, as well as every transported variable. The corresponding code listings can be found as supplementary material and in a public git repository.

Protocol	Initial Deployment		Migration	
	${\mathrm{LoC}}_{\mathrm{Pair}}$	${\mathrm{LoC}}_{\mathrm{Var}}$	${\mathrm{LoC}}_{\mathrm{Pair}}$	${\mathrm{LoC}}_{\mathrm{Var}}$
AMQP	67	2	48	0
Beckhoff ADS	81	2	62	0
Apache Kafka	74	2	55	0
MQTT	51	2	32	0
OPC UA	114	2	95	0
MEAN	77.4	2	58.4	0

**Table 3:** Specific modeling efforts $e_{i}$ per element measured for an experienced engineer. The corresponding model can be found as supplementary material.

	$e_{\mathrm{Pair}}$	$e_{\mathrm{Var}}$
Initial deployment	10 min	1 min
Migration	1 min	0

## Data Availability

The data presented in this study are available in the supplementary material and under https://gitlab.lrz.de/TUMWAIS/public/datacollectionarchitecture.modeldriven.
